# Breastfeeding Duration Is Associated With Domain-Specific Improvements in Cognitive Performance in 9–10-Year-Old Children

**DOI:** 10.3389/fpubh.2021.657422

**Published:** 2021-04-26

**Authors:** Daniel A. Lopez, John J. Foxe, Yunjiao Mao, Wesley K. Thompson, Hayley J. Martin, Edward G. Freedman

**Affiliations:** ^1^Division of Epidemiology, Department of Public Health Sciences, University of Rochester Medical Center, Rochester, NY, United States; ^2^The Cognitive Neurophysiology Laboratory, The Del Monte Institute for Neuroscience, Department of Neuroscience, University of Rochester Medical Center, Rochester, NY, United States; ^3^Division of Health Service Research and Policy, Department of Public Health Sciences, University of Rochester Medical Center, Rochester, NY, United States; ^4^Division of Biostatistics, Department of Family Medicine and Public Health, University of California, San Diego, CA, United States

**Keywords:** child, breastfeeding, neurocognition, cognitive development, public health

## Abstract

Significant immunological, physical and neurological benefits of breastfeeding in infancy are well-established, but to what extent these gains persist into later childhood remain uncertain. This study examines the association between breastfeeding duration and subsequent domain-specific cognitive performance in a diverse sample of 9–10-year-olds enrolled in the Adolescent Brain Cognitive Development (ABCD) Study®. The analyses included 9,116 children that attended baseline with their biological mother and had complete neurocognitive and breastfeeding data. Principal component analysis was conducted on data from an extensive battery of neurocognitive tests using varimax-rotation to extract a three-component model encompassing General Ability, Executive Functioning, and Memory. Propensity score weighting using generalized boosted modeling was applied to balance the distribution of observed covariates for children breastfed for 0, 1–6, 7–12, and more than 12 months. Propensity score-adjusted linear regression models revealed significant association between breastfeeding duration and performance on neurocognitive tests representing General Ability, but no evidence of a strong association with Executive Function or Memory. Benefits on General Ability ranged from a 0.109 (1–6 months) to 0.301 (>12 months) standardized beta coefficient difference compared to those not breastfed. Results indicate clear cognitive benefits of breastfeeding but that these do not generalize to all measured domains, with implications for public health policy as it pertains to nutrition during infancy.

## Introduction

The health benefits of breastfeeding for the mother and child are well-established. They include protection against infection for the child and reduced breast cancer risk for the mother (1). There is less agreement on whether breastfeeding improves cognitive performance in offspring, and whether certain cognitive domains are differentially impacted by breastfeeding. To date, there are only a few studies that have explored the association between breastfeeding duration and child cognitive performance without focusing entirely on intelligence quotient (IQ) and similar measures of general intellectual ability (2–4).

One explanation posited for the inconsistent results between breastfeeding and improvements in cognition is residual confounding resulting from non-experimental study designs (3). Previous research has noted that there are major social differences in women who choose to breastfeed their children. In the United States, significant disparities in breastfeeding outcomes are seen among individuals from different racial and socioeconomic backgrounds (5). For example, the 2011–2015 National Immunization Survey reported breastfeeding initiation rates of 64.3% for non-Hispanic black infants, and 81.5% for white infants (6). As a result, selection bias in observational studies has been a persistent criticism by those who dispute the association, particularly among studies with participation rates that are differential with respect to participant race or socioeconomic status. In addition, quasi-experimental methods such as propensity scoring have shown diminished associations of breastfeeding and cognitive performance that possibly reflect residual confounding due to the non-randomized design of observational studies (7).

Here, we focus on the association of breastfeeding duration on the following broad components: General Ability, Executive Function, and Memory, with special emphasis on controlling for confounding factors. General Ability is a measure of global intellect (8). Observational studies have been inconsistent in reporting a positive association between breastfeeding and improvements in General Ability. The United Kingdom Millennium Cohort Study, a study consisting of 11,879 term and preterm children, concluded that breastfed children were 1–6 months ahead in their cognitive development when compared to children never breastfed (9). Likewise, the British Avon Longitudinal Study of Parents and Children and the Brazilian Pelotas cohorts showed a strong positive association between breastfeeding and IQ scores, despite the two countries having vastly different breastfeeding patterns (i.e., in the UK breastfeeding is more common in middle/upper SES women, and in Brazil it is more common in lower SES women) (10). The only randomized control trial involving breastfeeding, conducted in Belarus with a sample of 13,889 newborns, concluded that breastfeeding had a significant positive association with IQ scores at age 6 (11). In contrast, a study using the Early Childhood Longitudinal Study Birth Cohort did not find a strong association between breastfeeding, math, and reading skills after adjustment for parent-child interaction (12). That study concluded that it was the characteristics of the mother, rather than the act of breastfeeding, that were the source of cognitive improvement.

Executive Function encompasses a broad range of behaviors that include planning, organization, impulse control, and goal-seeking (13). Executive function development begins in early childhood and can continue into adulthood (14). Developmental trajectories of executive function can be impacted by socioeconomic disparities and other early childhood adversities, such as preterm birth (15, 16). The few studies that examined the relationship between executive function and breastfeeding have had mixed conclusions. A study using 1,037 mother-child pairs in the Project Viva birth cohort did not find an association between breastfeeding duration and executive function in mid-childhood (median age = 7.7 years) (2). In contrast, a Spanish study using two birth-cohorts with 500 mother-child pairs reported a significant relationship between increased breastfeeding duration and increased executive functioning performance in 4-year-old children (17).

Memory is a broad construct that encompasses separate systems of information encoding, storage, and retrieval (18). Memory is a multifaceted cognitive domain that includes subdomains like declarative memory and episodic memory (19). In children and adolescents, the ability to recollect past events improves throughout the teenage years (20). The literature on breastfeeding and memory is nearly non-existent. The few studies that have reported results have found no associations. A study that examined the relationship between breastfeeding and memory in 6–8 year-old children that had very low birth weight did not detect a strong association (21). A separate assessment of breastfeeding and verbal memory at age 53 also did not find a strong association (22). In addition, the Project Viva birth cohort did not find any association between memory scores at age seven and breastfeeding duration (23).

The aims of the current study were 2-fold. First, we attempt to thoroughly address confounding introduced by the observational study design using a quasi-experimental method. Second, we examined the association between breastfeeding duration and separate components representing cognition. We hypothesized that greater periods of breastfeeding duration would be strongly associated with improved scores for all neurocognitive components in later childhood (ages 9–10). The study was conducted using data from the baseline visit of the ABCD Study® (Data Release 2.0.1).

## Materials and Methods

### Study Design and Sample

The ABCD Study enrolled 11,875 children aged 9–10 throughout the United States between 2016–2018. Children were enrolled using a stratified, probability sample of schools at 21 currently active study sites and are followed using yearly in-person visits along with semi-annual follow-up calls. The ongoing study will continue until the original cohort of children are at least 19–20 years old (24). The study was designed to approximate the sociodemographic sampling distribution of the American Community Survey (24). In addition, parents/guardians were enrolled alongside children and completed a series of questionnaires related to the youth's early developmental history (25). Parental consent and child assent was obtained from all ABCD participants (24).

For this cross-sectional analysis, we excluded children who did not attend the baseline visit with a biological mother to minimize the impact of measurement error related to breastfeeding duration and other observed covariates (e.g., prenatal alcohol exposure). More than 85% (*n* = 10,131) of ABCD participants attended the baseline with their biological mother. As a result, all covariates reflect the experience of the participating child or the biological mother (e.g., parental education). Participants missing breastfeeding information (*n* = 175) or any neurocognitive test (*n* = 854) were excluded from the analyses. The final analyses exploring the association between breastfeeding duration and neurocognitive performance were conducted using data from 9,116 children. This study follows the Strengthening the Reporting of Observational Studies in Epidemiology (STROBE) reporting guideline for cross-sectional studies.

### Breastfeeding Measures

Parents at baseline were asked whether the child had ever been breastfed, and for how many months the child had been breastfed. Duration of breastfeeding was originally measured in months and was operationalized as a categorical variable with four levels (0 months, 1–6 months, 7–12 months, and more than 12 months) to examine the relationship between length of breastfeeding and lack of exposure to breastfeeding. Categorical levels of breastfeeding duration were selected to reflect cut-off points that are both commonly reported in the literature and recommended in pediatric guidelines (3, 26). Additional consideration was also made to prevent creating subgroups that had small sample sizes that could increase standard errors and decrease precision (27). Breastfeeding exclusivity was not collected as part of the ABCD assessment.

### Neurocognitive Measures

Children completed a neurocognitive battery reflecting many facets of cognition during the baseline ABCD visit (28). The ABCD neurocognitive battery incorporated several measures from the NIH Toolbox®-Cognition battery, the Flanker Test, the Picture Sequence Memory Test, the List Sorting Working Memory Test, the Picture Vocabulary Test, the Oral Reading Recognition Test, the Dimensional Change Card Sort Test, and the Pattern Comparison Processing Speed Test. In addition, each child at baseline completed the Matrix Reasoning Test from the Wechsler Intelligence Scale for Children, the Rey Auditory Verbal Learning Test (RAVLT), the Little Man Task, and the Cash Choice Task (28). The RAVLT is used to assess short-term and long-term retention of information (28). For our analyses, the RAVLT was transformed into three variables, encompassing short-term, long-term, and working memory. The short-term component incorporated the first five trials of the RAVLT, which asked children to recall a list of 15 words immediately after each trial. The sixth trial involved recalling the original 15 words after a “distractor” list of 15 words was presented (28). The long-term memory component included only the seventh trial that was administered after a 30-min delay.

### Cognitive Outcomes

The latent structure of the cognitive tests was identified using principal component analysis (PCA) with varimax-rotation. PCA is a dimensionality reduction technique used to group highly correlated variables into fewer components to improve interpretation (29). PCA with varimax-rotation has previously been used with the ABCD baseline data to identify the latent dimensions of the administered neurocognitive tests (30). For our analyses, all neurocognitive tests were standardized with a mean of zero and a variance of one prior to PCA estimation due to the differing scales of the original measures. After the initial factor extraction, a varimax rotation was applied to maximize the shared variance of items within each principal component and improve interpretation of the results (31). An eigenvalue-one criterion was used to determine whether to retain a component (32). Factor loadings with a standardized estimate of 0.5 or higher were considered strong (33). Variables were considered for removal if they had significant cross-loading (e.g., 0.4 loading on more than one factor). Factor scores for each participant were then extracted to reflect that individual's performance across the separate components. The PCA with varimax-rotation was conducted using the **psych** package in R (34).

### Propensity Score Estimation and Diagnostics

The propensity score (PS) is an estimate of the probability of exposure to a treatment conditional on observed covariates (35). The purpose of the PS is to allow for an unbiased estimate of the treatment effect on an outcome when randomization (e.g., assigning breastfeeding exposure) is not feasible (36). For our analyses, PS weights were used to balance the distribution of confounding variables between each level of breastfeeding duration. The decision to use PS weights instead of a regression-based, covariate-adjustment technique (e.g., multinomial logistic regression) was due to multiple factors. First, a large number of variables can be summarized using PS techniques to minimize residual confounding in observational studies (37). Second, there were significant differences in the balance of certain participant characteristics between levels of breastfeeding duration (e.g., race/ethnicity). Relatedly, PS weighting can account for the unequal probability of participating in a study due to the characteristics of the participant (27). Third, PS weights reduce the risk of bias by estimating scores independent of the outcome (i.e., cognitive outcomes were excluded from the estimation of the PS weights).

The PS weights were estimated via generalized boosted modeling (GBM). GBM is a multivariate non-parametric regression technique that iteratively estimates the PS of individuals to maximize balance in observed covariates (38, 39). GBM can also incorporate interactions among a large number of covariates to reduce the risk of model misspecification (39, 40). Previous research has shown that GBM outperforms logistic regression in PS estimation when there is moderate non-additivity and moderate non-linearity in the model (27). GBM was selected for the current analyses to adequately address the risk of biased estimates resulting from model misspecification. In addition, GBM can be extended to treatments with more than two levels to improve covariate balance between different exposure groups (38). The mean outcomes of different treatment groups can then be compared to describe average treatment effects (e.g., the mean outcome had all children in the study been breastfed for 7–12 months compared with the mean outcome had all children in the study been breastfed for 1–6 months). The present analyses used a 30,000- tree GBM with an interaction depth of 4, a shrinkage of 0.001, and a bag fraction of 0.5 to optimize covariate balance and minimize extreme weights. Estimation of the PS for multiple treatments using observed covariates was conducted with the R-package **twang** (41).

Variables for the PS estimation were selected using a review of established selection criteria (42, 43). Child measures included gender, race/ethnicity, age at baseline, birth weight, child relationship in his/her family, family conflict, school risk and protective factors, weeks born premature, cesarean birth, and whether the child had other complications at birth (e.g., required oxygen, slow heartbeat, cyanosis, number of days in incubator). Maternal measures included age at birth of child, educational attainment and household income at baseline, tobacco and alcohol use during pregnancy, marital status at baseline, whether the pregnancy was planned, prenatal vitamin usage, and pregnancy-related diabetes. In addition, sampling weights provided in the ABCD dataset were included to account for underrepresentation or overrepresentation of certain subgroups (44).

Balance diagnostics for PS weights were included in the analyses according to established best practices (45). The diagnostics focused on two criteria for determining effective balancing: the *absolute standardized mean difference* (ASMD) of weighted and unweighted variables, and the range of weights to determine the presence of extreme weights. For our analysis we considered any ASMD >0.10 as a sign of covariate imbalance. Anything 0.10 or less has been suggested in the literature as a negligible difference in relative balance (36, 46). A form of doubly robust estimation further adjusted for any covariates that remained imbalanced to improve the accuracy of the estimation and minimize the mean square error (38, 47).

### Missing Data

The amount of missing data were <2% for all covariates included in the PS estimation except for days in incubator (3.3%) and household income (8.6%). Inverse probability weighting using GBM is considered an alternative to multiple imputation that is effective and requires fewer assumptions (48). GBM can account for missing data by creating a missing value indicator to avoid discarding of data (i.e., listwise deletion) (38). As a result, the reported estimates only utilize the non-imputed data and did not require the removal of any additional participants due to missing data.

### Sensitivity Analyses

Sensitivity analysis was important due to the lack of maternal neurocognitive testing in the ABCD Study. A method for quantifying vulnerability to unmeasured confounding was used that leveraged the strength of observed covariates to estimate the robustness of the research conclusions. Maternal education was selected to bound the strength of an unobserved confounder due to its high correlation with intelligence in the literature (49). The sensitivity analysis was conducted using the **Sensemakr** package in R (50).

An additional sensitivity analysis was conducted using multiple imputation by chained equations to explore the impact of missing neurocognitive data on the PCA loadings. The multiple imputation was conducted using the **mice** package in R (51).

### Analytic Strategy

Comparison of variable distributions used the mean for continuous variables and percentages for categorical variables. Significance testing used *t* tests for continuous variables and χ^2^ tests for categorical variables. After PS estimation, a doubly robust propensity-score adjusted linear regression model was used to estimate the association of breastfeeding duration on separate components. Results of the linear regression are presented as standardized beta coefficients to reflect the standardization of factor scores extracted from the PCA. All tests for statistical significance were two sided and considered statistically significant at *P* < 0.05. Standard errors and corresponding 95% confidence intervals are reported using bootstrapping with 1,000 replications. The regression was performed using the **Survey** package in R (52). All statistical analyses were performed using R version 3.6.2. R code for replication can be retrieved at https://git.io/JOIJJ.

## Results

### Demographic Characteristics of the Sample

The analytic sample included 9,116 children that attended the baseline evaluation with their biological mother and did not have missing breastfeeding information or cognitive test scores. As seen in Table 1, there was an imbalance in the characteristics of children across the different levels of breastfeeding duration. Breastfeeding duration was not significantly related to child's gender, cyanosis, slow heartbeat at birth, and school risk report (all *p*-values > 0.05). There were significant differences across breastfeeding duration for the remaining covariates (all *p*-values < 0.05). Of the 1,875 children never breastfed, 36.9% were White, 32.9% were Black and 19.3% were Hispanic. When compared to the other breastfeeding groups, a greater proportion of never breastfed children had unmarried mothers (52.5%), had mothers with a high school diploma or less (33.0%), and came from households with a combined income under $50,000 (43.9%).

**Table 1:** Characteristics of the ABCD Sample (*n* = 9,116^†^).

	**Duration of breastfeeding**				
	**0 months (*n* = 1,875)**	**1–6 months (*n* = 3,241)**	**7–12 months (*n* = 2,198)**	**>12 months (*n* = 1,802)**	***p*-Value^‡^**
Age at baseline, months	118.98 ± 7.28	119.39 ± 7.48	118.69 ± 7.56	118.47 ± 7.59	<0.001
Birth weight, oz	106.82 ± 22.99	109.40 ± 24.19	115.19 ± 22.59	118.80 ± 20.89	<0.001
Maternal age at birth of child	27.05 ± 6.42	29.02 ± 6.19	30.50 ± 5.54	31.10 ± 5.51	<0.001
Weeks born premature	1.2 ± 2.47	1.2 ± 2.48	0.7 ± 1.87	0.5 ± 1.58	<0.001
Child's sex, *n* (%)					0.723
Male	947 (50.5)	1,684 (52.0)	1,145 (52.1)	935 (51.9)	
Female	928 (49.5)	1,557 (48.0)	1,053 (47.9)	866 (48.1)	
Child's race/ethnicity, n (%)					<0.001
White	692 (36.9)	1,608 (49.6)	1,387 (63.1)	1,188 (65.9)	
Black	617 (32.9)	455 (14.0)	158 (7.2)	86 (4.8)	
Hispanic	361 (19.3)	772 (23.8)	393 (17.9)	319 (17.7)	
Asian	13 (0.7)	47 (1.5)	46 (2.1)	35 (1.9)	
Other	192 (10.2)	354 (10.9)	210 (9.6)	174 (9.7)	
Household income in dollars, *n* (%)					<0.001
<50,000	824 (43.9)	914 (28.2)	403 (18.3)	324 (18.0)	
50,000–99,999	431 (23.0)	832 (25.7)	579 (26.3)	513 (28.5)	
100,000–199,999	287 (15.3)	886 (27.3)	749 (34.1)	641 (35.6)	
≥200,000	88 (4.7)	318 (9.8)	326 (14.8)	224 (12.4)	
Maternal educational level, *n* (%)					<0.001
< High school diploma	221 (11.8)	177 (5.5)	91 (4.1)	60 (3.3)	
HS Diploma/GED	398 (21.2)	320 (9.9)	122 (5.6)	89 (4.9)	
Some College	743 (39.6)	1,124 (34.7)	517 (23.5)	367 (20.4)	
Bachelor	300 (16.0)	897 (27.7)	779 (35.4)	627 (34.8)	
Post Graduate Degree	211 (11.3)	714 (22.0)	688 (31.3)	659 (36.6)	
Marital status, *n* (%)					<0.001
Married	858 (45.8)	2,114 (65.2)	1,748 (79.5)	1,411 (78.3)	
Not Married	984 (52.5)	1,095 (33.8)	441 (20.1)	386 (21.4)	
Tobacco use during pregnancy, *n* (%)					<0.001
No	1,660 (88.5)	3,078 (95.0)	2,168 (98.6)	1,774 (98.4)	
Yes	213 (11.4)	157 (4.8)	30 (1.4)	27 (1.5)	
Alcohol use during pregnancy, *n* (%)					<0.001
No	1,858 (99.1)	3,174 (97.9)	2,134 (97.1)	1,744 (96.8)	
Yes	17 (0.9)	59 (1.8)	60 (2.7)	54 (3.0)	
Planned pregnancy, *n* (%)					<0.001
No	1,051 (56.1)	1,346 (41.5)	593 (27.0)	485 (26.9)	
Yes	810 (43.2)	1,880 (58.0)	1,592 (72.4)	1,310 (72.7)	
Cesarean section, *n* (%)					<0.001
No	1,061 (56.6)	1,850 (57.1)	1,475 (67.1)	1,242 (68.9)	
Yes	811 (43.3)	1,391 (42.9)	721 (32.8)	560 (31.1)	
Prenatal vitamins, *n* (%)					<0.001
No	135 (7.2)	119 (3.7)	58 (2.6)	54 (3.0)	
Yes	1,711 (91.3)	3,089 (95.3)	2,121 (96.5)	1,728 (95.9)	
Gestational diabetes, *n* (%)					<0.001
No	1,704 (90.9)	2,982 (92.0)	2,075 (94.4)	1,689 (93.7)	
Yes	160 (8.5)	254 (7.8)	121 (5.5)	106 (5.9)	
Required oxygen at birth, *n* (%)					<0.001
No	1,675 (89.3)	2,808 (86.6)	1,987 (90.4)	1,642 (91.1)	
Yes	180 (9.6)	396 (12.2)	193 (8.8)	145 (8.0)	
Cyanosis, *n* (%)					0.476
No	1,796 (95.8)	3,082 (95.1)	2,108 (95.9)	1,723 (95.6)	
Yes	59 (3.1)	116 (3.6)	62 (2.8)	59 (3.3)	
Slow heartbeat at birth, *n* (%)					0.678
No	1,790 (95.5)	3,110 (96.0)	2,113 (96.1)	1,726 (95.8)	
Yes	60 (3.2)	94 (2.9)	60 (2.8)	46 (2.6)	
Relationship, *n* (%)					<0.001
Singleton	1,174 (62.6)	2,068 (63.8)	1,564 (71.2)	1,400 (77.7)	
Sibling	227 (12.1)	412 (12.7)	333 (15.2)	217 (12.0)	
Twin	466 (24.9)	748 (23.1)	301 (13.7)	185 (10.3)	
Triplet	8 (0.4)	13 (0.4)	0 (0)	0 (0)	
Days in Incubator	1.5 ± 5.7	1.5 ± 5.1	1.0 ± 4.9	0.6 ± 3.0	<0.001
Family conflict (youth reported)	2.4 ± 2.1	2.1 ± 1.9	1.9 ± 1.9	1.9 ± 1.9	<0.001
School risk (youth reported)	20.0 ± 3.0	19.9 ± 2.8	19.9 ± 2.6	19.9 ± 2.8	0.940

*Data are presented as mean (SD), except where noted*.

† *Table includes only those who were not missing breastfeeding and neurocognitive data*.

‡ *t-test for continuous covariates and chi-square test for categorical covariates*.

A separate comparison of children missing neurocognitive data (Table 2) revealed significant differences only for race/ethnicity (*p* = 0.03), household income (*p* = 0.02), maternal education (*p* < 0.01), school risk report (*p* < 0.01), and mother's marital status (*p* < 0.01). A sensitivity analysis using multiple imputation of the missing neurocognitive tests did not reveal any changes to the PCA loadings (Supplementary Table 2). Characteristics of the full sample without exclusion for those that did not attend baseline with their biological mother also did not reveal any significant differences in the distribution of important covariates (Supplementary Table 3).

**Table 2:** Characteristics of participants missing a neurocognitive test.

	**Missing neurocognitive test**		
**Characteristic**	**No (*n* = 9,277)**	**Yes (*n* = 854)**	***p*-Value^†^**
Age at baseline, months	118.97 ± 7.48	118.88 ± 7.25	0.737
Birth weight, oz	112.05 ± 23.44	112.89 ± 23.15	0.320
Maternal age at birth of child	29.36 ± 6.14	29.50 ± 6.23	0.510
Weeks born premature	0.95 ± 2.22	0.88 ± 2.23	0.396
Child's sex, *n* (%)			0.636
Male	4,790 (51.6)	454 (53.2)	
Female	4,485 (48.4)	400 (46.8)	
Child's race/ethnicity, *n* (%)			0.034
White	4,898 (52.8)	417 (48.8)	
Black	1,377 (14.8)	133 (15.6)	
Hispanic	1,903 (20.5)	207 (24.2)	
Asian	148 (1.6)	6 (0.7)	
Other	942 (10.2)	90 (10.5)	
Household income in dollars, *n* (%)			0.016
<50,000	2,557 (30.2)	274 (35.6)	
50,000–99,999	2,371 (28.0)	215 (27.9)	
100,000–199,999	2,572 (30.4)	205 (26.6)	
≥200,000	957 (11.3)	76 (9.9)	
Maternal educational level, *n* (%)			<0.001
<High school diploma	600 (6.5)	89 (10.4)	
HS Diploma/GED	973 (10.5)	99 (11.6)	
Some College	2,785 (30.0)	244 (28.6)	
Bachelor	2,630 (28.3)	226 (26.5)	
Post Graduate Degree	2,277 (24.5)	194 (22.7)	
Marital status, *n* (%)			<0.001
Married	6,194 (67.4)	519 (61.1)	
Not Married	2,999 (32.6)	330 (38.9)	
Tobacco use during pregnancy, *n* (%)			0.390
No	8,830 (95.3)	819 (95.9)	
Yes	433 (4.7)	35 (4.1)	
Alcohol use during pregnancy, *n* (%)			0.630
No	9,067 (97.9)	833 (97.9)	
Yes	192 (2.1)	18 (2.1)	
Planned pregnancy, *n* (%)			0.260
No	3,557 (38.3)	347 (40.6)	
Yes	5,660 (61.0)	504 (59.0)	
Missing	60 (0.6)	3 (0.4)	
Cesarean section, *n* (%)			
No	5,720 (61.7)	522 (61.1)	0.490
Yes	3,548 (38.2)	330 (38.6)	
Gestational diabetes, *n* (%)			0.830
No	8,592 (92.9)	786 (92.4)	
Yes	654 (7.0)	65 (7.6)	
Required oxygen at birth, *n* (%)			0.480
No	8,253 (89.9)	771 (90.0)	
Yes	929 (10.1)	76 (9.0)	
Prenatal vitamins, *n* (%)			0.170
No	385 (4.2)	37 (4.4)	
Yes	8,784 (95.8)	813 (95.6)	
Relationship, *n* (%)			0.710
Singleton	6,326 (68.2)	588 (68.9)	
Sibling	1,209 (13.0)	117 (13.7)	
Twin	1,721 (18.6)	148 (17.3)	
Triplet	21 (0.2)	1 (0.1)	
Days in Incubator	1.18 ± 4.9	1.17 ± 5.9	0.970
Family conflict (youth reported)	2.05 ± 1.96	2.09 ± 1.94	0.624
School risk (youth reported)	19.94 ± 2.82	19.64 ± 3.08	0.003

*Data are presented as mean (SD), except where noted*.

† *t-test for continuous covariates and chi-square test for categorical covariates*.

### Results of the Principal Component Analysis

Factor loadings (Table 3) supported the extraction of three components from the PCA. The resulting three-component model excluded the Cash Choice Task because it loaded separately into a fourth factor and did not meaningfully improve the variance explained by the PCA. The three-component model was further supported by the scree plot showing eigenvalues above 1 only for the first three components (Supplementary Figure 1). There was no evidence of significant cross-loadings for any of the variables included in the PCA. The three-component model explained 59.2% of the cumulative variance. Total variance around 60% is typically considered satisfactory due to the imprecise nature of data when dealing with human populations (33). General Ability loadings consisted of the List Sorting Working Memory test, the Oral Reading Recognition test, the Picture Vocabulary test, the Matrix Reasoning test, and the Little Man Task. The extracted General Ability scores ranged from −3.75 to 3.68 (median = 0.02). Executive Function loadings consisted of the Flanker test, the Dimensional Change Card Sort test, and the Pattern Comparison Processing Speed test. Executive Function scores ranged from −3.97 to 2.75 (median = 0.06). The Memory component included all three RAVLT measures, along with the Picture Sequence Memory test. Memory scores ranged from −4.14 to 3.36 (median = 0.04).

**Table 3:** Principal component loadings with varimax-rotation for three-component model (*n* = 9,116)^†^.

	**General ability**	**Executive function**	**Memory**
Flanker test	0.23	**0.71**	0.08
List sorting working memory test	**0.59**	0.20	0.29
Dimensional change card sort test	0.25	**0.71**	0.19
Pattern comparison processing speed test	0.03	**0.80**	0.11
Picture sequence memory test	0.24	0.21	**0.52**
Oral reading recognition test	**0.75**	0.12	0.17
Picture vocabulary test	**0.74**	0.09	0.18
Matrix reasoning test	**0.64**	0.08	0.20
Little man task	**0.52**	0.28	0.08
Rey auditory verbal learning test–trial I–V	0.27	0.13	**0.83**
Rey auditory verbal learning test–trial VI	0.17	0.09	**0.89**
Rey auditory verbal learning test–trial VII	0.18	0.08	**0.89**

† *Cash-Choice Task excluded based on Kaiser rule. Bold values 0.50 or greater is considered practically significant*.

### Balance Diagnostics for the Propensity Score Estimation

**Figure 1:**
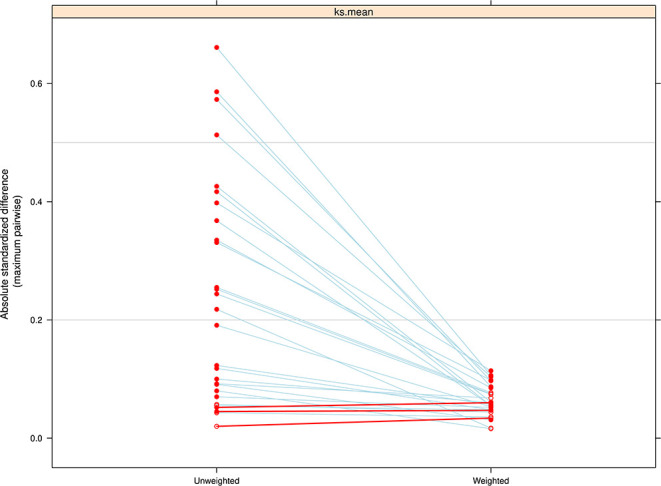
Comparison of the absolute standardized mean difference (ASMD) between treatment groups (levels of breastfeeding duration) on pretreatment covariates, before and after weighting. The weighting was successful in reducing imbalance across all covariates included in the model except for three that had a slight decrease in balance.

The PS estimation improved the balance in potential confounding variables for nearly every covariate included in the model (Figure 1). Despite improvements in balancing there was evidence of a modest imbalance for race/ethnicity (ASMD = 0.13) and tobacco use during pregnancy (ASMD = 0.12). These variables were further adjusted for in the final weighted linear regression model to minimize bias.

The presence of extreme weights in PS estimation can increase the variance of association estimates, although there is no clear cutoff for what constitutes an extreme weight (53). The distribution of weights in this study (range of 0.10–8.80) suggested a modest amount of imbalance, although the large sample size and the absence of weights equal to 0 or >10 indicate that the weights obtained are not extreme (53). As a result, the reported analysis did not use *ad-hoc* methods like weight trimming or weight stabilization.

PS estimation requires sufficient “common support” between treatment groups to ensure adequate covariate balance (53). In our analyses, there was some visual evidence of a lack of common support between those breastfed for 0 months and those breastfed for more than 12 months, but the lack of overlap was not extreme (Supplementary Figure 1). In addition, the absence of weights with a value of zero and weights with extreme values suggests that common support was not an issue in achieving covariate balance (53).

### Breastfeeding Duration Associated With General Ability

The PS-adjusted linear regression model found a strong association between breastfeeding duration and performance on General Ability scores (Table 4). The comparison was greatest when comparing those breastfed for more than 12 months to those never breastfed (β = 0.328; 95% CI, 0.238–0.418; *p* = 1.68 × 10^−12^). The estimate was reduced after additional adjustment of covariates that remained imbalanced (β = 0.301; 95% CI, 0.217–0.385; *p* = 4.04 × 10^−12^). The results suggest that being breastfed for more than 12 months is associated with an increase of about 0.30 standard deviations (SD) in General Ability scores. The association was also strong for those breastfed 7–12 months (β = 0.224; 95% CI, 0.147–0.301; *p* = 1.57 × 10^−8^) and 1–6 months (β = 0.109; 95% CI, 0.039–0.179; *p* = 0.002) relative to those not breastfed.

**Table 4:** Association between breastfeeding duration and domain-specific neurocognitive performance (*n* = 9,116)^†^.

	**General ability**		**Executive function**		**Memory**	
	**β (95% CI)**	***p-*Value**	**β (95% CI)**	***p-*Value**	**β (95% CI)**	***p-*Value**
Breastfeeding duration						
0 months (*n* = 1,875)	reference		reference		reference	
1–6 months (*n* = 3,241)	0.109 (0.039, 0.179)	0.002	−0.016 (−0.086, 0.054)	0.654	−0.031 (−0.108, 0.046)	0.427
7–12 months (*n* = 2,198)	0.224 (0.147, 0.301)	1.57 × 10^−8^	−0.001 (−0.078, 0.075)	0.972	−0.001 (−0.083, 0.081)	0.976
>12 months (*n* = 1,802)	0.301 (0.217, 0.385)	4.04 × 10^−12^	−0.017 (−0.101, 0.066)	0.684	−0.008 (−0.097, 0.080)	0.851

† *Propensity score-adjusted linear regression model with additional adjustment for imbalance covariates: race/ethnicity and tobacco use during pregnancy*.

### Breastfeeding Duration Is Not Associated With Executive Function

There was no indication that breastfeeding duration had an association with Executive Function (Table 4). Executive Function performance was lowest in those breastfed for more than 12 months, but the strength of the association was small and not statistically significant (β = −0.017; 95% CI, −0.101 to 0.066; *p* = 0.684). Executive Function scores were also lower for every level of breastfeeding duration relative to those not breastfed, although the estimated associations were consistently insignificant and likely not meaningful.

### Breastfeeding Duration Is Not Associated With Memory

There was also no indication that breastfeeding had any meaningful impact on Memory scores. Like Executive Function, Memory scores were lower for every level of breastfeeding duration relative to those not breastfed, but the associations at each level were small and not significant.

### Results of Sensitivity Analyses

Sensitivity analysis was conducted to gauge the robustness of the General Ability regression estimates (Supplementary Table 1). The analysis used a robustness value (RV) to summarize the resilience of the point estimates taking into account the partial R-squared of the treatment with the outcome (54). The RV was greatest in those breastfed more than 12 months (10%), indicating that any unmeasured confounder that cannot explain at least 10% of the residual variance in both the treatment and the outcome is insufficient to meaningfully change our conclusion (50). The RV is diminished but is still strong for 7–12 months (7.4%) and 1–6 months (5.1%). Finally, the contour plots (Supplementary Figure 2) show that a confounder with more than three times the strength of maternal education would be required to fully explain away our results.

An additional sensitivity analysis was conducted to examine the associations without a propensity model. A generalized linear model (GLM) was specified using the full cohort sample (*n* = 11,875) and adjustment for the same variables included in the PS model. It should be noted that multiple imputation was not used to account for missing data. As a result, the final sample size was 9,681 when using GLM. The results of the analysis (Supplementary Table 4) were almost identical to those found using PS weighting. Estimates were slightly attenuated for General Ability when using a GLM, although the differences are likely trivial. The associations for Executive Function and Memory remained small and not significant in the GLM results.

## Discussion

A goal of this study was to analyze the association between breastfeeding duration and cognitive performance in 9–10-year-old children. Compared to those never breastfed, General Ability performance was highest in those breastfed more than 12 months after weighting and adjustment for any remaining imbalanced covariates. The association of breastfeeding for more than 12 months with General Ability scores was about three-tenths of a SD, and about two-tenths of a SD when comparing children breastfed for seven to 12 months to those not breastfed. The estimate was reduced to about one-tenth of a SD in children breastfed for only 1–6 months. There was no statistically significant association between breastfeeding duration and Executive Function or Memory scores. Note that this improvement in General Ability persists and is being assessed up to a decade after cessation of breastfeeding. Future analyses using the ABCD dataset can illuminate whether this association remains or is weakened as children enter adolescence.

The strengths of this study include a large, diverse study sample with measurement of many potential confounders and predictors of both breastfeeding initiation and cognitive performance. The use of inverse probability weights to achieve a balance in observed covariates further decreases the chance that residual confounding influenced our results. The results of the sensitivity analysis demonstrated the robustness of the conclusions to unmeasured confounding and described the potential impact on biasing effect estimates.

There are potential limitations in our study. First, whether the effect of an unmeasured confounder like maternal IQ is plausibly more than twice as strong as maternal education cannot be answered using the ABCD dataset. In an analysis using the Project Viva cohort, the association estimates of the model without maternal IQ decreased by about 17% after adjustment for maternal IQ (from β = 0.35 to β = 0.29, respectively) (23). In addition, a meta-analysis assessing 18 studies found that adjustment for maternal IQ did not change the overall conclusion that breastfeeding was associated with higher performance in IQ tests (4). Second, by excluding those with missing neurocognitive data we may have biased our effect estimates, although results from a sensitivity analysis using multiple imputation on missing neurocognitive tests did not reveal any evidence of bias on our outcome measures. Third, while breastfeeding recall has been found to be reliable (55), it is important to keep in mind that ABCD parents were asked to recall information that concluded for some nearly a decade earlier. A separate study in Norway found that the majority of women, even after 20 years, were able to accurately recall breastfeeding duration within 1 month (56). In addition, any misclassification in maternal recall of breastfeeding duration is non-differential with respect to cognitive performance (i.e., exposure error is independent of the outcome) and is likely to bias the effect estimate toward the null.

Breast milk is widely recognized as an important contributor to healthy brain development. Previous research on nutrients in breast milk and postnatal cognitive development has focused on the role of arachidonic acid (ARA) and docosahexaenoic acid (DHA) (57). DHA is a long-chain omega-3 fatty acid that is produced by the mother and transferred to the fetus during the third trimester of pregnancy (57). After birth, breast milk is the primary source of DHA for infants, and DHA concentrations in breast milk reflect the mother's nutritional intake (58). DHA is directly implicated in the myelination of brain frontal lobes throughout childhood and adolescence (59). Disruptions in myelination trajectories due to poor nutrition (e.g., DHA deficiency) have previously been associated with poor cognitive outcomes (60). The reasons for a differential impact of breastfeeding on cognitive domains are not entirely clear. An analysis of maternal milk DHA values and performance on math scores in 28 countries reported a strong association (β = 0.462, *p* = 0.006) after adjustment for socio-economic influences and macronutrients (61). The researchers concluded that maternal milk DHA accounted for more variance in math scores than socio-economic factors. A separate study in Ghana found a positive but insignificant association between DHA and executive function in a sample of 307 2–6-year-old children (β = 0.25, *p* = 0.06) (62). A review of 15 studies evaluating DHA supplementation in children and cognitive outcomes did not find a consistent association between intake and memory scores (59). The researchers concluded that increases in DHA generally resulted in improved learning and behavioral outcomes in children (59).

Current policy recommends women breastfeed children through at least age 1 or as long as mutually desired (63). The results of our analysis support this recommendation to breastfeed for at least 1 year or longer to achieve full cognitive benefits, although whether the improvement on cognitive performance has any practical importance in the real world is not clear (64). One analysis using three separate cohorts in the United States found a significant economic gain in children who were breastfed in terms of income gained as an adult (65). Finally, from a public health perspective, small effects can have a large impact when the exposure is common (66). Breastfeeding is a common exposure that is not considered harmful, with numerous benefits for both mother and infant. In that sense, the recommendation of breastfeeding for longer durations can make a large impact on population health.

In conclusion, the current study identified a strong association between breastfeeding duration and General Ability scores, with the greatest effect found in those breastfed for more than 12 months. Sensitivity analysis showed that results were robust to unmeasured confounding, although the effect is substantially diminished under certain circumstances. We did not detect an association between breastfeeding duration and either Executive Function or Memory scores. Our findings suggest that the benefits of breastfeeding on General Ability are evident in offspring with only a few months of exposure and appear to increase past 12 months of exposure. The effect sizes are more meaningful when viewed from a population health perspective to measure the potential positive impact to society.

## Data Availability Statement

The datasets generated for this study can be found in online repositories. The names of the repository/repositories and accession number(s) can be found below: The ABCD Study data are openly available to qualified researchers for free. Access can be requested at https://nda.nih.gov/abcd/request-access. Code for replication of the analyses conducted in this article can be retrieved at https://git.io/JOIJJ.

## Ethics Statement

The studies involving human participants were reviewed and approved by Office for Human Subject Protection, University of Rochester Medical Center. Written informed consent to participate in this study was provided by the participants' legal guardian/next of kin.

## Author Contributions

DL conceived of the presented idea. DL and YM performed the computations. WT, YM, and HM verified the analytical methods. JF and EF encouraged DL and supervised the findings of this work. All authors discussed the results and contributed to the final manuscript.

## Conflict of Interest

The authors declare that the research was conducted in the absence of any commercial or financial relationships that could be construed as a potential conflict of interest.

## Abbreviations

ABCD: Adolescent Brain Cognitive Development
PCA: Principal Component Analysis
GBM: Generalized Boosted Model
ASMD: Absolute Standardized Mean Difference
RV: Robustness Value
RAVLT: Rey Auditory Verbal Learning Test
CI: Confidence Interval
GLM: Generalized Linear Model.
