# Supplementary material for: Breastfeeding Duration Is Associated With Domain-Specific Improvements in Cognitive Performance in 9–10-Year-Old Children
Source: Front Public Health. 2021 Apr 26;9:657422. doi: 10.3389/fpubh.2021.657422 (PMC8109433; doi:10.3389/fpubh.2021.657422)

Supplementary Material


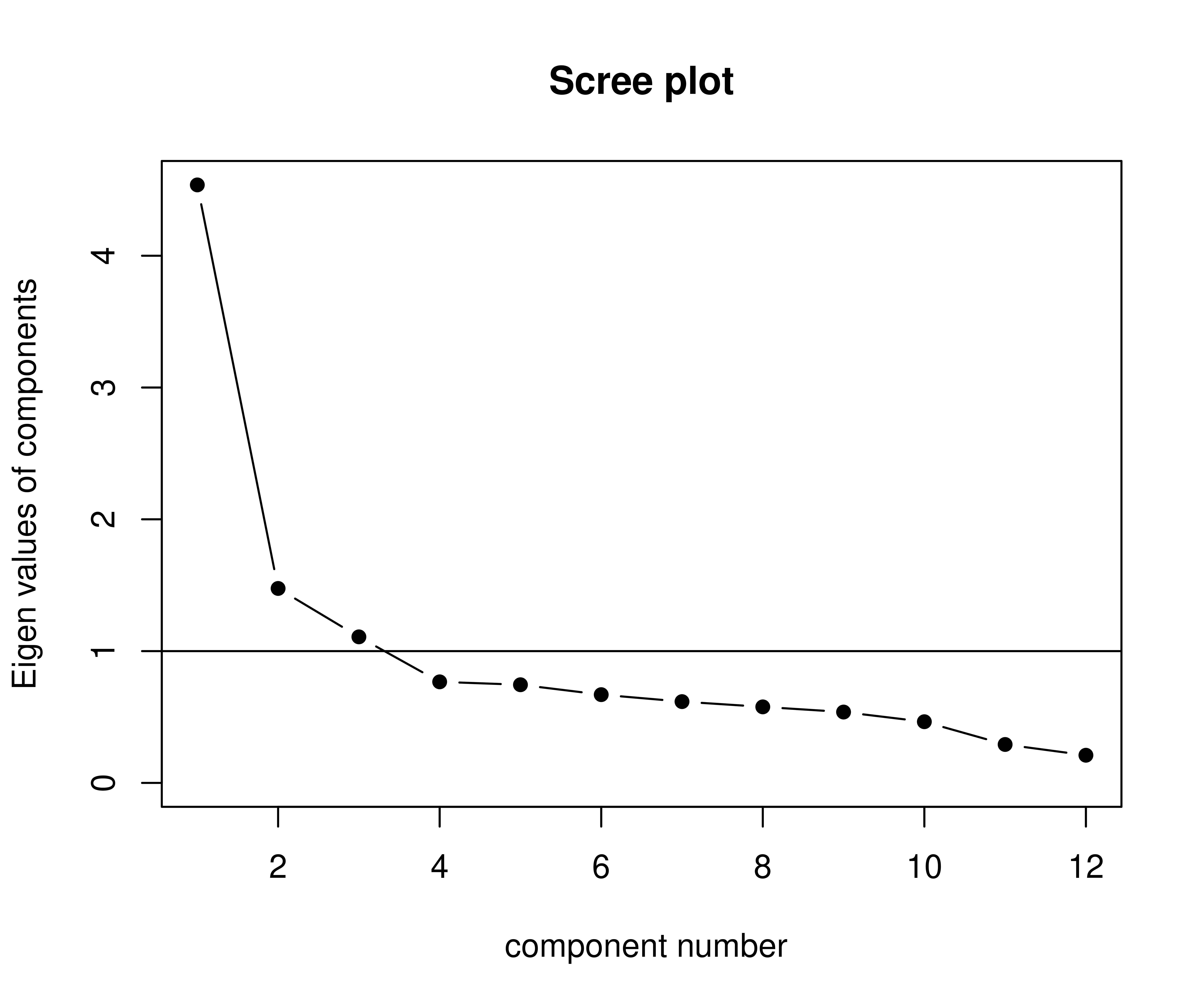


**FIGURE S1.** Scree plot showing the relationship between eigenvalue and number of components. Results of the scree plot show that a three-component model is optimal due to eigenvalues leveling off after three components.





**FIGURE S2.** Overlap of the empirical propensity score distributions – showing the spread of the estimated propensity scores in the treatment groups. Common support is important when comparing treatment groups (levels of breastfeeding duration).

**TABLE S1.** Sensitivity to Unobserved Confounders in the Association Between Breastfeeding Duration and General Ability


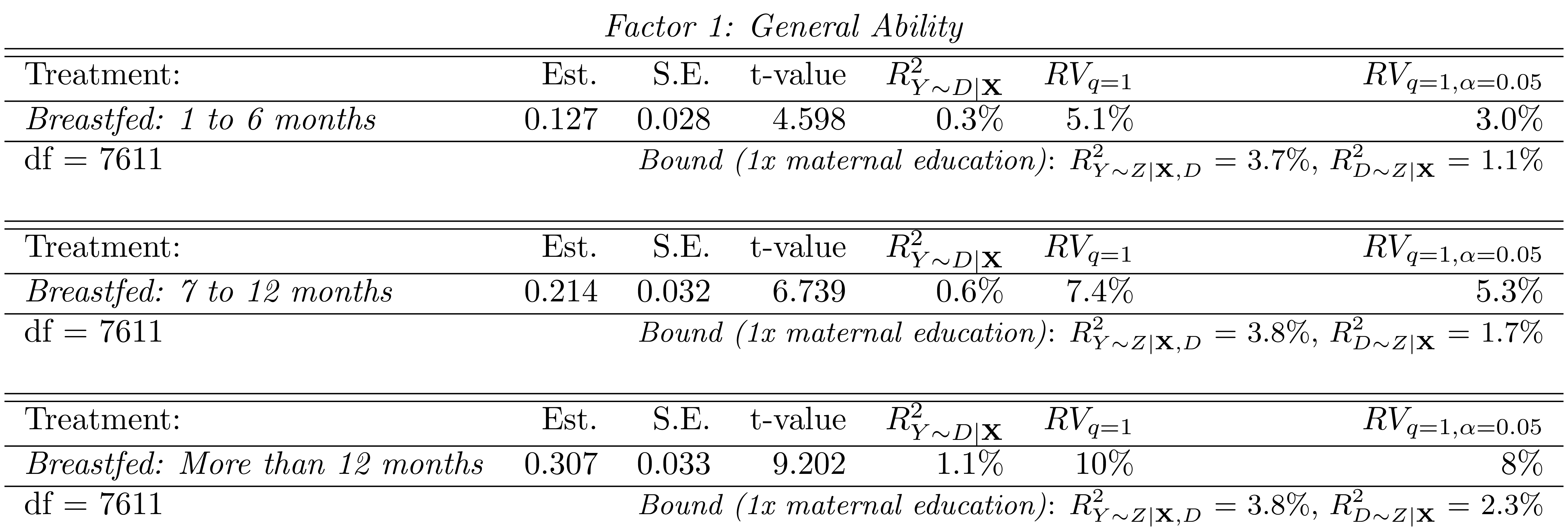





**FIGURE S3.** Contour levels represent the adjusted estimates of the exposure association (more than 12 months breastfed) on General Ability. The dashed line represents the necessary strength to change our conclusions (i.e., when the effect size includes the null value).

**TABLE S2.** Principal Component Loadings with Varimax-Rotation After Using Multiple Imputation for Missing Neurocognitive Tests (n=10131)


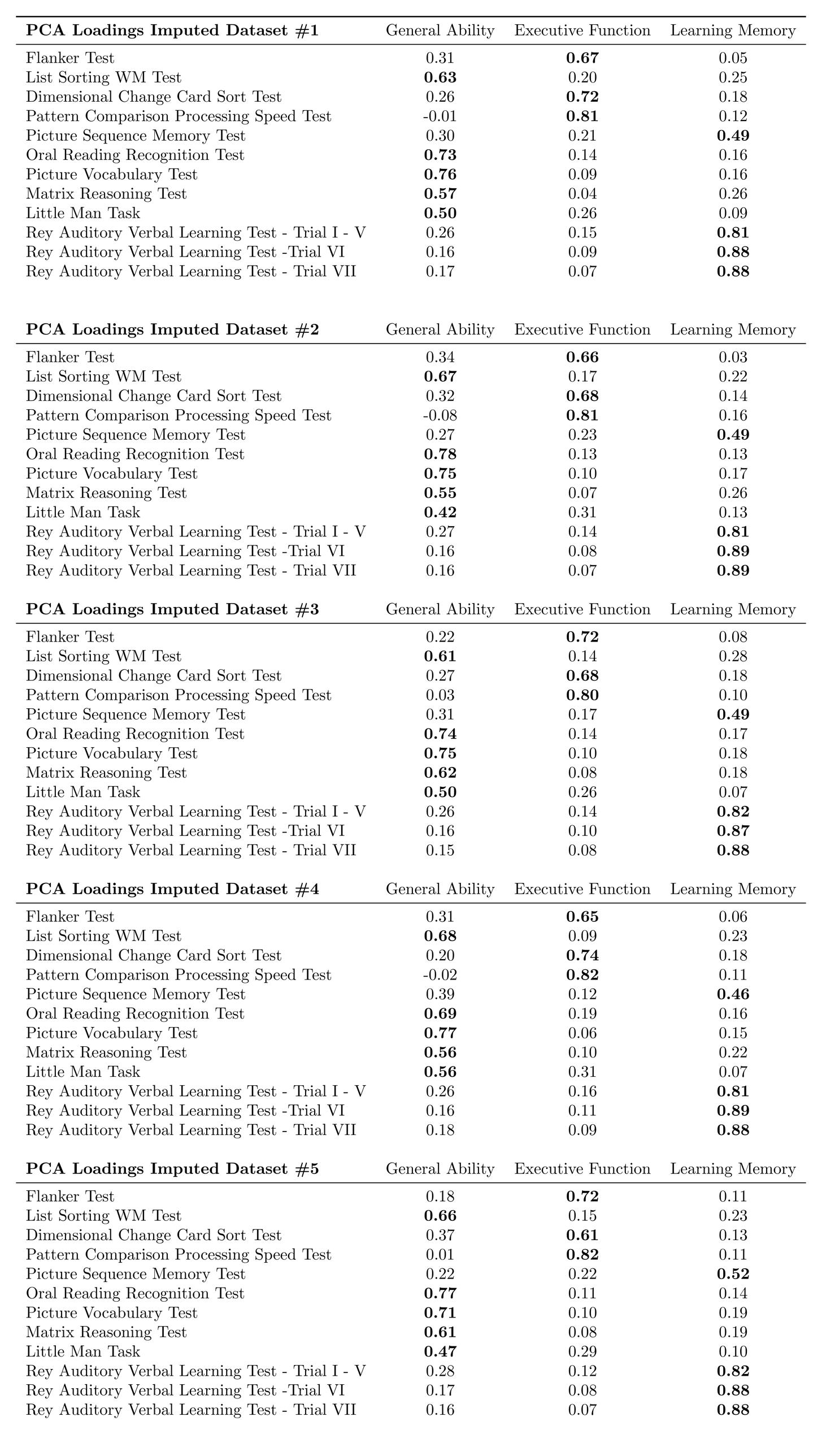


**Table S3.** Characteristics of the ABCD Sample by Duration of Breastfeeding Without Exclusion for Children Attending Baseline Without Biological Mother (n=11433*)


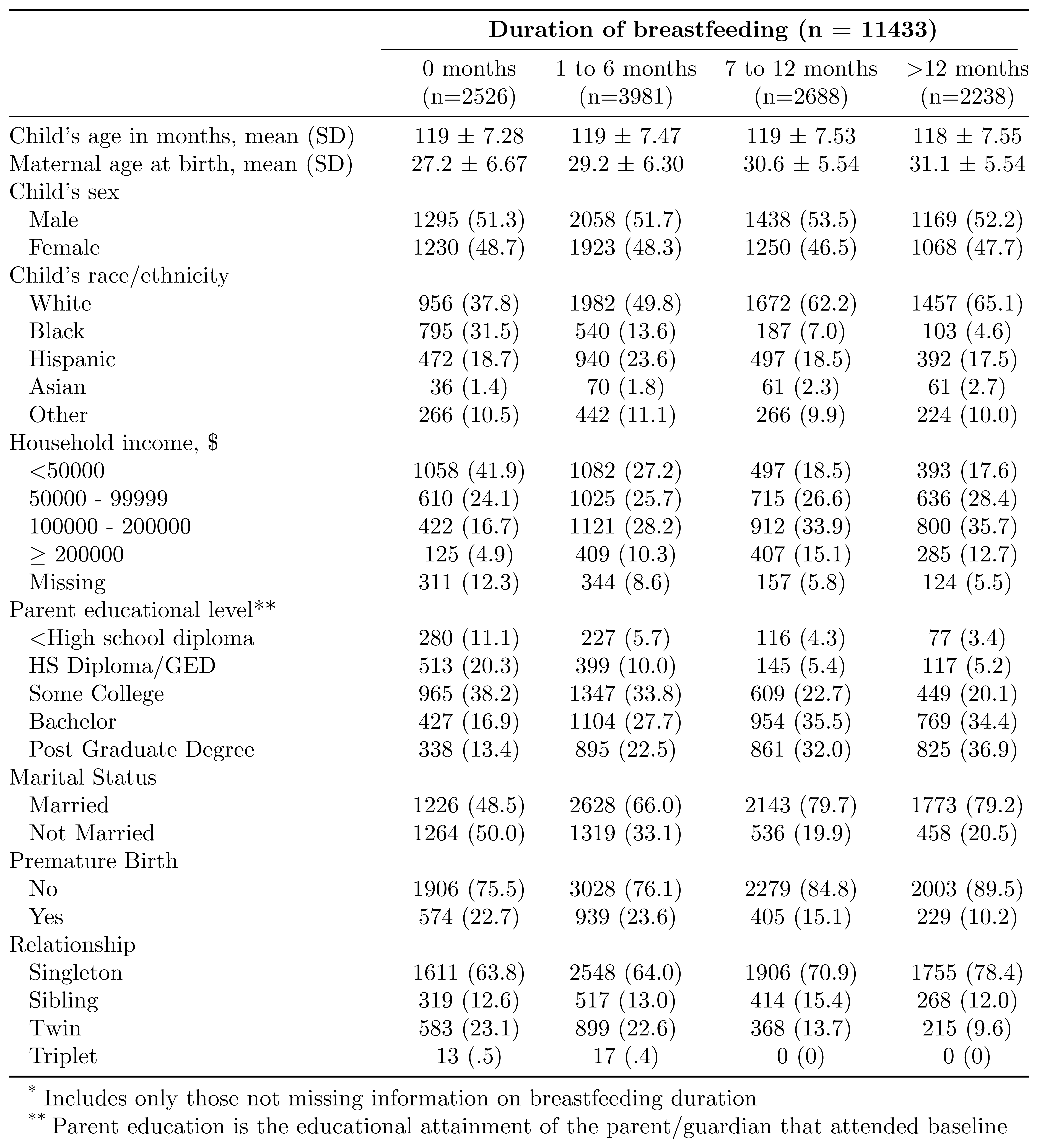


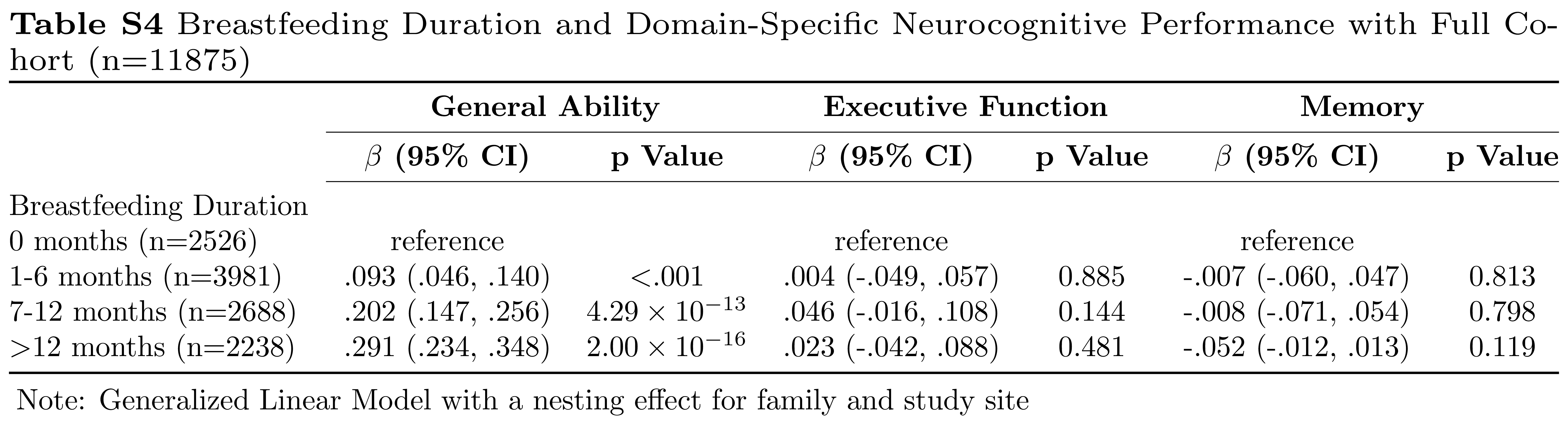

Supplement: Supplementary file 1 [file Data_Sheet_1.docx]
